# Burnout and quality of life in Portuguese healthcare professionals working in oncology and palliative care—a preliminary study

**DOI:** 10.1186/s12904-023-01273-7

**Published:** 2023-10-13

**Authors:** Florbela Gonçalves, Margarida Gaudêncio

**Affiliations:** 1 Faculty of Health Sciences, University Beira Interior, Covilhã, Portugal; 2 Internal Medicine and Palliative Care Service, Portuguese Institute of Oncology Francisco Gentil Coimbra, Coimbra, Portugal; 3 Faculty of Medicine, University of Coimbra, Coimbra, Portugal

**Keywords:** Burnout, Professional, Palliative care, Quality of life, Health professions

## Abstract

**Background:**

Palliative care is an approach that improves the quality of life of patients and their families who are facing challenges associated with life-threatening illness, through the prevention and relief of suffering. Palliative care health professionals are considered a risk group for the development of burnout, since they live with severe disease and death, on a daily basis. With this work, the authors intend to evaluate the quality of life and risk of b*urnout* in a group of health professionals, who work in a tertiary hospital dedicated to cancer patients.

**Material and methods:**

The authors conducted a quantitative, descriptive, correlational and transversal study on palliative care professionals working with cancer patients. The evaluation protocol used to collect data included a sociodemographic questionnaire, WHO Quality of life Assessment instrument and Maslach Burnout Inventory. Statistical analysis was performed using the SPSS®Statistics program.

**Results:**

In the sample, there is a predominance of female gender (79,4%) with a mean age of 43,2 ± 10,8 years. The most representative professional group was nursing (47,1%). The sample response rate was 91.9%. Analyzing Maslach Burnout Inventory score, it appears that physicians and nurses have higher levels of exhaustion when compared to the other groups. In relation to quality of life (QoL), it was observed that in all dimensions, there was a homogeneous distribution of responses. It was verified that it was not possible to establish any relationship between the dimensions of burnout and QoL. Thus, the various dimensions behaved independently.

**Discussion:**

Physicians and nurses had the highest burnout levels in the most dimensions of Burnout score, in which they were followed by the operational assistants, who had moderate scores. Despite hight prevalence of Burnout, there is no correlation between Burnout and quality of life in this population. The perception of QoL is very satisfactory in the sample studied may result from the fact that these individuals have developed adequate self-protection strategies, thus preventing QoL from being affected by Burnout.

**Conclusion:**

Prevention, diagnosis and intervention at burnout level is an important measure to be taken in health organizations, since the consequences that come from the experiences experienced by professionals will be reflected both in the quality of services provided to patients and in the QoL and well-being of professionals. Interventions are needed to promote better coping mechanisms when dealing with stress in this population. After this study, a Burnout Consultation was created at the Institution, to support professionals at risk or already affected.

**Supplementary Information:**

The online version contains supplementary material available at 10.1186/s12904-023-01273-7.

## Background/introduction

The philosophy and practice of Palliative Care (PC) requires a holistic approach of the patient and the acquisition of a set of emotional, communication and relational skills, as well as, resilience [1, 2].

In 1975, Jean Watson introduced the human caring theory, defining health in different domains—physical, mental and social well-being [3]. According to Watson, care includes looking for the multiple dimensions of human health, trying to practice a supportive, meaningful and compassionate care [4].

For PC professionals, the work of caring for patient with serious and complex illness can put their own well-being at risk [1, 2]. So, these health professionals are therefore one of the professional groups that most face chronic occupational stress, and for this reason more susceptible to developing Burnout syndrome [1, 2].

*Burnout* is, therefore, a syndrome that is defined by the conjugation of three dimensions: *Emotional exhaustion* (inability to make or give more of himself); *Depersonalization* (reflected in professional and personal relationships, becoming the coldest and most distant individuals) and *Low Personal Achievement* (which leads to loss of self-esteem and motivation, progressing to feeling of inadequacy and failure) [1, 2].

By the negative impact on workers’ health, organizations and society in general, burnout syndrome can be considered as a public health problem, which can happen in any profession [5]. However, it is recognized that it can be particularly evident in health professionals, given direct contact with people in distress, excessive work and emotional involvement in the patients’ problems, often in an environment of stress and conflict [5].

The presence of *burnout*can have harmful consequences on professional and personal life, affecting the quality of life (QoL) of the health care provider [4, 5]. In recent years there has been a growing interest in this topic, which is, currently, considered a disease [6, 7].

Given the complexity and individual diversity of variables in QoL, there is still no consensus on its definition [8]. The concept of QoL is used in different situations and contexts, extending to all sectors of society [8]. There is no universally accepted definition of quality of life, as it is a multidimensional concept [9]. More recently, with the aim of clarifying this concept, World Health Organization (WHO) debated this issue and defined quality of life as an individual’s perception of their position in life, within the context of the culture and value systems in which they live [10]. So, quality of life involves objective dimensions (living conditions, professional situation and salary) and subjective dimensions (well-being, family, love and personal fulfillment) [9].

The current situation of change in organizations, particularly in health services, where work overload, frequent changes and conflicts in teams contribute to insecurity and instability [8]. So, these situations can lead to attitudes of resilience or risk to the well-being and QoL of individuals [8].

Regarding the impact of the working conditions in burnout and QoL of health professionals, the studies that have been done in this area revealed that a non-ethical work environment can correlate with the burnout triad [11].

The relationship between health care providers and patients is also affected [12–14]. The patients and families reported lower satisfaction with the quality of care and support provided by emotionally exhausted and unmotivated professionals [12–14]. There is also a negative impact on the quality of life of professionals [12–14].

Another important aspect to consider is the impact of shift work [15]. Some authors observed that shift work may have direct repercussions on personal and family life, since the number of weekly work hours and the way they are distributed can lead to burnout and, consequently, impair QoL [15].

Palliative care professionals are permanently subject to numerous situations of great emotional demand, either by contact with patients or by the work environment itself [16].

In 2017, 43.9% of United States (US) physicians reported burnout and the prevalence of burnout symptoms across different specialties ranged from 29.6% (occupational medicine) to 54.9% (emergency medicine) [17]. In palliative care physicians, the burnout prevalence have been reported as 32 to 35% [18]. In other countries, the burnout prevalence in palliative care have been reported from 24% (Australia) to 41.9% (Singapore) [19, 20]. Burnout syndrome has a high prevalence among nurses [21]. This healthcare group of professionals is one of the most affected, with sociodemographic (gender, age, marital status), occupational (level of healthcare, work shift and healthcare service areas), psychological factors (neuroticism, agreeability, extraversion and conscientiousness) that can influence its development [21].

In recent years, the interest in study the QoL and risk of burnout in these professional has been arisen, since they constitute a vulnerable group, consequence of the potential chronic stress experienced [16].

Exploring the literature, it appears that in Portugal, there are some studies of burnout in physicians and nurses from different areas [22–25]. Most of the studies have been performed during the COVID-19 pandemic. So, there are some gaps remaining, particularly in the oncology palliative care. In order to address this gap, we conducted a study to better understand the prevalence of burnout in this population and to examine the relationship between burnout, demographic and job factors, but also, quality of life (QoL).

The aim of the study was to evaluate the burnout and QoL of cancer palliative health care professionals.

## Material and methods

The authors conducted a quantitative, descriptive, correlational and transversal study on palliative care professionals working in Portugal in a specific illness context.

### Sample

The authors used a convenience sample consisting of 34 health care providers working in the context of cancer palliative care. Regarding the inclusion and exclusion criteria in the study, the authors consider all professionals who worked in the Palliative Care Unit in a tertiary hospital dedicated to cancer patient.

The sample had included 3 physicians, 16 nurses, 2 hospital secretaries; 1 psychologist; 1 social worker and 2 volunteers. This study included professionals who agreed to participated, were able to understand the purpose of the study and gave their consent to participate.

### Data collection

Data collection respected the rules of the Helsinki Protocol and the Oviedo Convention, and it was been approved by the ethics and management committee of the hospital.

The data measurement tools were distributed individually, accompanied by a letter explaining the nature and aims of the study and ensuring the confidentiality of the data.

After authorization by informed consent, the data were collected through an evaluation protocol designed for this purpose. The protocol included a sociodemographic questionnaire (*age, gender, marital status, schooling, professional category, years of work, weekly workload, night work, employment link, management position, sleep hours per day*), a QoL assessment questionnaire (WHO Abbreviated Quality of Life Assessment Instrument, WHOQOL-Bref) and a burnout level assessment questionnaire (Maslach Burnout Inventory, MBI, authorized by Mind Garden®), which ranks in high, medium or low, the possible scores.

The Maslach Burnout Inventory (MBI) consists of three dimensions (Emotional Exhaustion, Depersonalization, Personal Accomplishment), defined taking into account different items (Supplementary Data) [26]. The 22 items were recorded as assuming a value between 0 and 6 (Supplementary Data) [26]. There is no recoding of these values. The calculation of the scores for each of the dimensions uses these values [26]. The burnout scale can either be evaluated using continuous values that vary between 0 and 54 in Emotional Exhaustion dimension, 0 and 30 in Depersonalization dimension, 0 and 48 in the Personal Accomplishment dimension (characterized by mean, standard deviation, medians, quartiles and extreme values) [26].

It is also possible to define three levels of burnout in each dimension: Emotional Exhaustion (Low: score ≤ 18; Medium: score 19–26; High: score ≥ 27); Depersonalization (Low: score ≤ 5; Medium: score 6–9; High: score ≥ 10); Personal Accomplishment (Low: score ≤ 33; Medium: score 34–39; High: score ≥ 40) [26]. In this case, burnout is defined as a combination of high levels of emotional exhaustion and depersonalization and low levels of personal accomplishment [27].

The WHOQOL-Bref is a subjective and multidimensional scale designed to assess the quality of life of healthy or unhealthy individuals. It consists of 26 questions (2 of them are related to general aspects; 24 questions are related to specific aspects and organized into four domains: physical, psychological, social relations and environment) [28] (Supplementary Data). For each of the domains, the score varies between 0 and 100, or between 4 and 20, with higher values corresponding to a better perception of quality of life [28]. In the study, the authors used the score between 4 and 20.

### Statistical analysis

Before analysis, the database was anonymized. In the sample, the observed variables were characterized taking account the most adequate descriptive statistics.

Categorical and qualitative variables were expressed with absolute and relative frequencies (N and %). Quantitative/continuous variables were characterized by the mean and standard deviation.

Quantitative and qualitative variables were compared with Kruskal–Wallis test.

Before determining Cronbach's alpha indexes [29], the descriptive statistics of each item observed at each scale were determined, namely the mean and the standard deviation. It was necessary to look at the correlation coefficients between each two items of the same dimension. We opted for the Kendall's Tau-b coefficient since each item only takes up to five different values.

Pestana and Gageiro recommend the following criteria for Cronbach's alpha: values above 0.80 are desirable; higher than 0.70 are recommended; and greater than 0.60 should be accepted for research use only [30]. Thus, within the framework of this study, any result greater than 0.60 represents satisfactory internal consistency.

The Cronbach's coefficient for the total of 22 items in the MBI scale is given by (α = 0.688) and for the total of 26 items in the WHOQOL Bref scale is given by (α = 0.879). These results indicate satisfactory and desirable indices of internal consistency for each scale respectively.

The database was organized in Microsoft® Excel® program. Statistical analysis was performed using the SPSS®Statistics program (version 21.0 for Windows®, IBM®). The tests were performed at a significance level of 5% (*p* < 0.05).

## Results

### Sociodemographic characteristics

The sample response rate was 91,9%. The general sociodemographic characteristics of the sample are described in Table 1.

**Table 1:** Sociodemographic characteristics of the sample

VARIABLES		SAMPLE
**Age (years)**	Median	43.2
	Standard deviation	10.8
**Weekly workload**	Median	39.2
	Standard deviation	5.7
**Gender, n (%)**	Male	7 (20.6)
	Female	27 (79.4)
**Marital status, n (%)**	Single	6 (17.6)
	Divorced	3 (8.8)
	Widow	2 (5.9)
	Married	23 (67.6)
**Schooling (years), n (%)**	≤ 4 years	1 (2.9)
	5–9 years	1 (2.9)
	10–12 years	8 (23.5)
	> 12 years	24 (70.7)
**Professional category, n (%)**	Physician	3 (8.8)
	Nurse	16 (47.1)
	Nurse assistant	9 (26.5)
	Others^*^	6 (17.6)
**Years of work, n (%)**	< 3 years	5 (14.7)
	3–5 years	1 (2.9)
	6–10 years	7 (20.6)
	> 10 years	21 (61.8)
**Night work, n (%)**	Yes	23 (67.6)
	No	11 (32.4)
**Employment link, n (%)**	Yes	29 (85.3)
	No	5 (14.7)
**Management position**	Yes	5 (14.7)
	No	29 (85.3)
**Sleep hours per day, n (%)**	< 6 hours	10 (29.4)
	6–8 hours	24 (70.6)

^*^2hospital secretaries; 1 psychologist; 1 social worker and 2 volunteers

In the sample, there is a predominance of female gender (79.4%) with a mean age of 43.2 ± 10.8 years. Most of the professionals are married (67.6%) and have advanced education (70.5%). The most representative professional group was nursing (47.1%).

Regarding the work characteristics, it was found that the professionals worked 39.2 ± 5.7 h per week. The generality of the population worked at night (67.6%). Most of them had an employment link with the institution (85.3%), where they had worked for more than 10 years (61.8%), and did not hold any management position (85.3%).

### Quality of life and burnout assessment

**Fig. 1:**
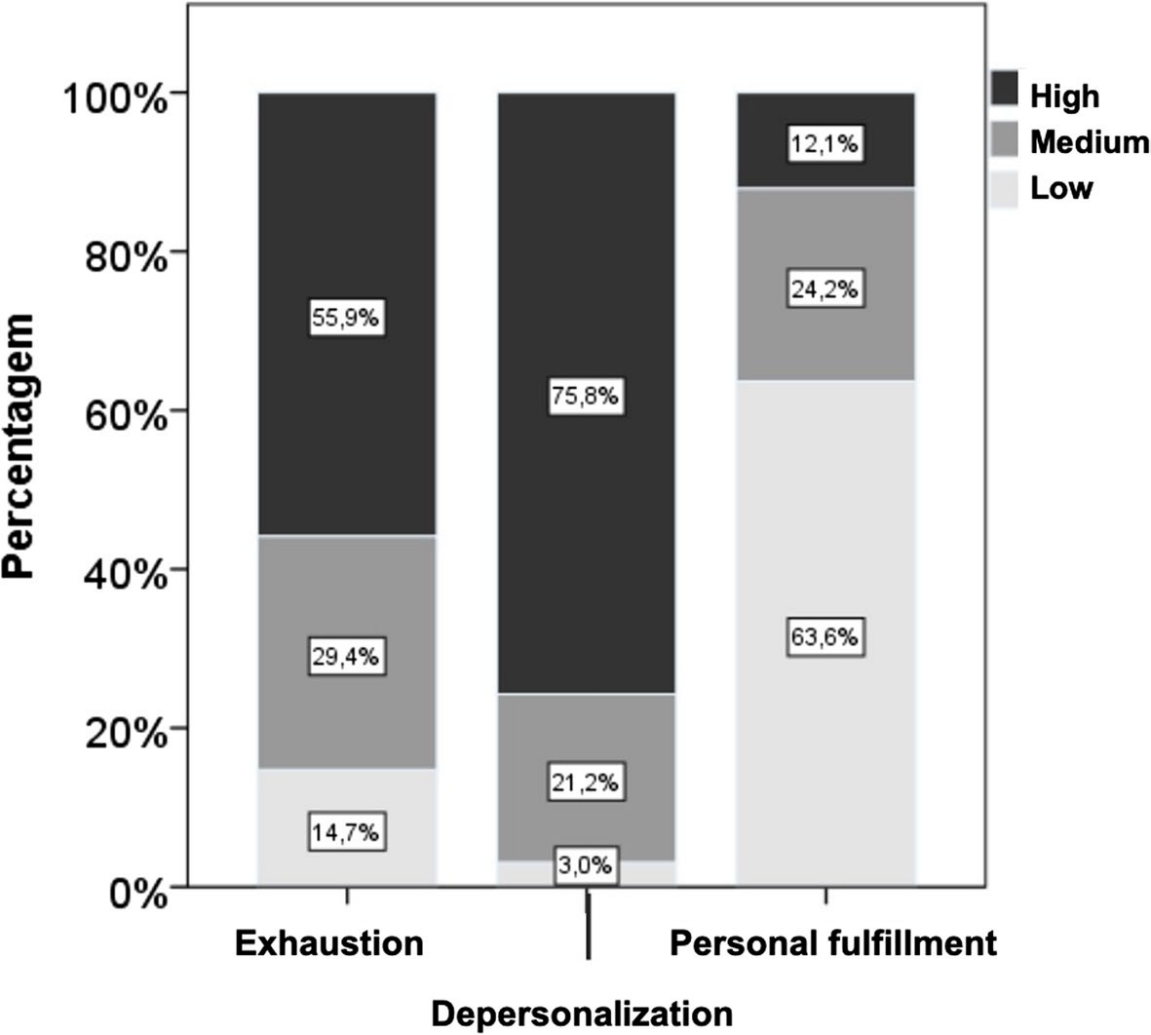
Burnout dimensions and their predominance in the sample according to the MBI scale score

Figure 1 and Tables 2 - 3 show the results of the different burnout dimensions, according to the Maslach Burnout Inventory (MBI) score.

**Table 2:** Burnout dimensions in the sample according MBI score (mean and standard deviation)

	***Emotional Exhaustion***	***Depersonalization***	***Personal Achievement***
Mean	22.2	6.9	33.5
N	34	34	34
Standard deviation	13.3	4.0	7.0

**Table 3:** The three dimensions of the Burnout survey, MBI, for each of the groups of health professionals (mean ± standard deviation)

	***Emotional Exhaustion***	***Depersonalization***	***Personal Achievement***
Physician	25.0 (± 22.9)	6.0 (± 4.3)	35.3 (± 16.1)
Nurse	26.7 (± 11.9)	9.0 (± 4.5)	32,9 (± 4.5)
Nurse assistant	18.9 (± 9.9)	6.0 (± 3.0)	32.9 (± 6.0)
Other^a^	12.5 (± 8.2)	3.8 (± 1.9)	34.7 (± 9.9)
*P value*	*0.102*	***0.03***	*0.715*

^*a*^* 2 hospital secretaries; 1 psychologist; 1 social worker and 2 volunteers*

As can be seen in Fig. 1, for the dimension *“Exhaustion"*, 55.9% of the professionals presented high levels, 29.4% medium levels and 14.7% low levels. In the dimension *“Depersonalization”,* the differences between the levels were more relevant in the sample, where 75.8% presented high levels, 21.2% medium levels and only 3% low levels. In the dimension *"Personal achievement/fulfillment”,* the majority of individuals (63.6%) presented a low score (less than 33–34), translating a high negative perception of personal achievement.

In the sample, there is a mean score of 22.2 (± 13.3) in the “Exhaustion” dimension, 6.9 (± 4) in. “ Depersonalization” and 33.5 (± 7) in “ Personal Achievement” dimension (Table 2).

Analyzing Table 3, it appears that physicians and nurses have higher levels of exhaustion when compared to the other groups. Regarding the “ Personal Achievement” dimension, there are practically no differences between the groups. However, in the “Depersonalization” dimension, there are statistically significant differences. The nurses have higher levels of depersonalization, when compared to other groups.

**Fig. 2:**
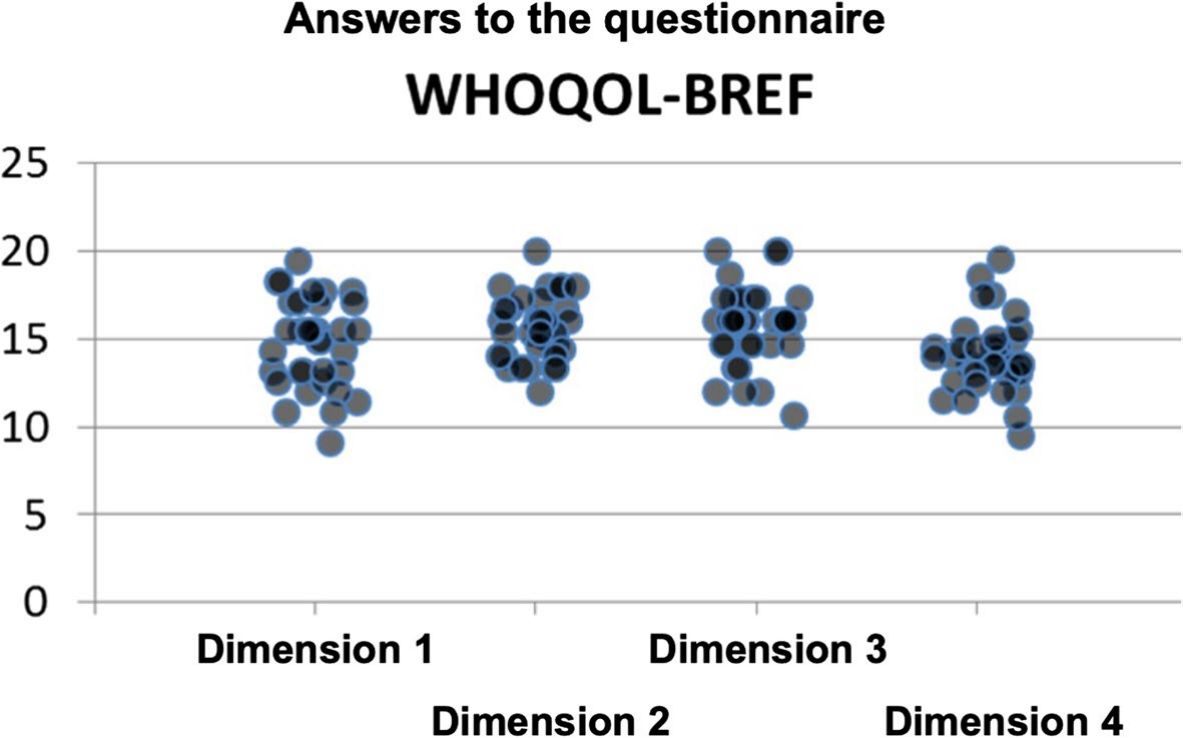
Scatter diagram—distribution of responses in the different dimensions of QoL

In relation to quality of life (QoL), it was observed that in all dimensions, there was a homogeneous distribution of responses. In Fig. 2 and Table 4, we find the results of the individually obtained responses, which presented a very satisfactory score in the various dimensions of the QoL.

**Table 4:** The dimensions of Quality of Life (QoL) (mean and standard deviation)

	***Physical***	***Psychological***	***Social Relations***	***Environment***
Mean	14.7	15.6	15.7	14.0
N	34	34	34	34
Standard deviation	2.5	1.8	2.2	2.1

In the sample, there is a mean score of 14.7 (± 2.5) in the Physical dimension of QoL, 15.6 (± 1.8) in Psychological dimension, 15.7 (± 2.2) in Social relation dimension and 14.0 (± 2.1) in Environment (Table 4).

Analyzing Table 5, it appears that physicians and others have higher levels of quality of life in physical dimension. In psychological and social relations’ dimension, there are similar results between the professional groups, except the other’s group, that present the higher scores. Finally, in the environment’s dimension, nurses have lower levels of quality of life, when compared with the other professional groups. Although, these results were no statistical significants (*p* > 0.05).

**Table 5:** The dimensions of QoL scores for each of the groups of health professionals (mean ± standard deviation)

	***Physical***	***Psychological***	***Social Relations***	***Environment***
Physician	15.43 (± 2.29)	15.56 (± 2.34)	15.11 (± 1.54)	15 (± 3.28)
Nurse	14.36 (± 2.59)	15.41 (± 1.67)	15.42 (± 2.57)	13.81 (± 2.1)
Nurse assistant	14.60 (± 2.71)	15.56 (± 2.29)	15.85 (± 2.35)	14 (± 2.25)
Other^a^	15.62 (± 2.52)	16.29 (± 1.45)	16.89 (± 1.09)	14.18 (± 1.95)
*P value*	*0.655*	*0.726*	*0.338*	*0.9*

^*a*^*2hospital secretaries; 1 psychologist; 1 social worker and 2 volunteers*

By analyzing Table 6, it was verified that it was not possible to establish any relationship between the dimensions of burnout and the dimensions of QoL studied. Thus, the various dimensions behaved independently and do not present any statistical differences. Although there was a high risk of burnout in the sample studied, the results suggest that, in a relatively homogeneous way, individuals had a good perception of their QoL in each of the dimensions, in particular.

**Table 6:** Presence of Burnout and perception of quality of life

Variables	N	Physical	Psychological	Social Relations	Environment
**Emotional Exhaustion**					
Low	5	13.9 (± 3.1)	15,1 (± 2,1)	15,7 (± 1.1)	14,3 (± 2.7)
Medium	10	15.7 (± 1.8)	15,9 (± 1.6)	16,5 (± 2.2)	14,6 (± 1.4)
High	19	19 (± 2.5)	15,3 (± 1.9)	15,3 (± 2.4)	13,6 (± 2.3)
*** P value***	-	0.250	0.621	0.3	0.279
**Depersonalization**					
Low	1	15.4	13.3	12.0	13.0
Medium	7	14.8 (± 2.3)	15.8 (± 1.6)	16.5 (± 1.0)	15.0 (± 2.0)
High	25	14.7 (± 2.6)	15.6 (± 1.9)	15.6 (± 2.4)	13.7 (± 2.2)
*** P value***	-	0.944	0.374	0.173	0.268
**Personal achievement**					
Low	21	14.8 (± 2.2)	15.5 (± 1.5)	15.9 (± 2.2)	14.2 (± 2.2)
Medium	8	14.1 (± 3.2)	16.0 (± 2.5)	16.1 (± 2.4)	13.6 (± 2.6)
High	4	14.4 (± 2.3)	14.3 (± 1.3)	14.0 (± 1.7)	13.7 (± 1.5)
*** P value***	-	0.753	0.289	0.186	0.472

## Discussion

Palliative care (PC), carried out by a multidisciplinary team, should be provided on the basis of patients' needs, such as the high suffering associated with the disease, and not on the basis of diagnosis [31].

Working in a specialized service as oncological PC, has specificities and needs that impose and require professionals an increased dedication and effort, compared to other health contexts [31].

So, these professionals are exposed to pain and suffering, as well as complex and advanced health problems, sometimes with difficult decisions to make, namely with ethical implications [31].

For this reason, these professionals constitute a population more vulnerable to Burnout syndrome [31]. Burnout syndrome is related to experiences of stress at work, when the individual is faced with a mismatch between expectations and personal / professional motivations, and the resources that work offers to satisfy them [31].

In the present study, the authors tried to evaluate the risk of burnout and quality of life of health professionals working in cancer PC, in hospital context.

After the evaluation of the dimensions of Burnout, it was observed that most of professionals in this study presented moderate to high levels of "Emotional exhaustion" and “Depersonalization”, as well as, low levels of "Personal achievement". So, at the time of the study, these professionals presented high risk of Burnout. These results, although the sample is small, are in agreement with the literature.

According to the different professional groups, it was found that, for the dimension "Emotional exhaustion”, higher levels were identified in physicians and nurses. In the group of nurse assistant, the levels of this dimension were low to moderate. In the group designated as “others" (that included social worker and psychologists) the levels were low.

For the dimension “Depersonalization”, only the group “others" showed low levels, while physicians, nurses and nurse assistants had moderate levels. However, the group of nurses presented the highest scores, with statistical significance, compared to the others. In the dimension "Personal achievement”, there were small differences between professional groups (physicians – moderate levels; nurses—moderate to high levels; nurse assistants- high levels; “other” – moderate levels).

Gómez—Urquiza et al. analize the levels and prevalence of burnout’s dimensions in PC nurses [32]. The results of this review and meta-analysis have shown that between 24 and 30% of PC nurses were suffering one of the burnout dimensions [32]. “Depersonalization” was the most affected dimension, because working conditions were harsh and it ends up producing exhaustion and inefficiency with the patient [32].

The results indicate that in the different dimensions of burnout in the group of PC professionals, the scores are relatively high in emotional exhaustion (55.9% had a high score). This can be explained by the fact that, PC professionals are exposed to crisis situations as the transmission of bad news, confrontation with death and suffering, dealing with ambivalent families and the need to deal with their own emotions to help others.

In this study, relatively high levels of depersonalization (75.8%) and low to moderate levels of personal achievement (63.6%) were also observed. Both Metha and Garcia et al. found that nurses working in PC had a reduced personal achievement [33–35]. However, Emold et al. observed that, about 80% of health professionals in cancer units had very satisfactory levels of personal fulfillment [34].

In the study, there was a trend of similar levels in the dimensions of burnout between physicians, nurses and nurse assistants. However, in the group “others”, there was an inverse trend (low emotional exhaustion and depersonalization score and moderate personal achievement).

We know that the practice of medicine, particularly in this area, can provide opportunities to develop a career with huge meaning and satisfaction [35–38]. Taking into account the service provided, which requires a different involvement with work and with patients, especially because of the life/death limit situation they experience, it is important to design strategies capable of promoting the development of personal and situational resources capable of facilitating stress management, minimizing their effect on the health status of individuals and their work. These strategies may include meditation and mindfulness, training of effective communication techniques and self-care [39, 40].

Regarding QoL, the results showed that, this was not influenced by the high burnout rates. Although 19 individuals in the sample presented a high burnout score in the dimension "Emotional exhaustion", the perception of quality of life in the different dimensions varied between 13.6 and 19 (scale of 4–20). Regarding "Depersonalization", 25 individuals presented a high score, but their perception of QoL in the various dimensions was from 13.7 to 15.6 on the same scale. In the "Personal achievement", 21 of the elements presented a low score (i.e., a low personal achievement), but the level of QoL perception varied between 14.2 and 15.9. The same trend was observed in quality of life in general (4, i.e., Good) and in health-related conditions in which most individuals felt quite satisfied.

These results may seem contradictory. However, it can be explained by the fact that, these professionals are part of a cohesive team, with solid personal relationships. Furthermore, they have a long and extensive care experience, which may have facilitated the development of mechanisms of inter-help and coping, and thus prevent the high levels of Burnout, experienced by professionals, have a significant impact on QoL.

Pereira et al. developed a study in physicians and nurses who worked at palliative care units in Portugal [25]. This study showed a low risk of burnout (55%). These results were similar with the literature [41, 42].

Palliative medicine professionals are characterized by resilience, indispensable to face the challenges that are naturally associated with this area. Although curative therapeutic success is fruitful, "terminality" is naturally accepted, enjoying "small big" successes, such as controlled intense pain, a quiet and suffering-free death, reconciliation. Positive reinforcement of third parties (friends and family of patients) is part of the daily life of this team and helps make your path easier [43, 44].

Pereira et al. identified some protective factors to burnout, as religious and spiritual dimension, but also, training in palliative care [43, 44].

In this sample, there was a satisfactory perception of QoL. This means that these professionals have already developed the appropriate self-protection strategies, thus preventing their QoL from being affected by burnout. Thus, prevention, diagnosis and intervention at burnout level is an important measure to be taken in health organizations, since the consequences that come from the experiences experienced by professionals will be reflected both in the quality of services provided to patients and in the QoL and well-being of professionals.

The authors recognize some limitations of the present research. One of the limitations was the sample size. However, according to internal consistency, it is a robust sample. This study was carried out in the context of a PC team. Therefore, the sample is so heterogeneous, just like other teams in this area, which must be multidisciplinary. On the other hand, the presence of greater representation of certain professional groups allowed reaching more conclusions.

On the other hand, the fact that this study took place only in one institution, limited the sample size and the type of PC patients observed. The patients in palliative care observed in this study are only oncological patients, not considering non-cancer patients. This fact was explained by the target population of the hospital, where the study took place.

Further studies are needed to complement these results, for example, in the context of hospice and PC community teams. In the near future, it would be interesting to extend this study to other national units.

## Conclusion

This study evaluated the quality of life and risk of burnout in cancer palliative health care professionals.

Our findings indicate that, there was no significant differences in quality of life and risk of burnout between professional groups. According to the different dimensions of burnout, it was observed that most of professionals in this study presented high risk of burnout, in agreement with literature.

Burnout leads to absenteeism, ineffective communication, medical errors and job abandonment [43, 44]. Interventions are needed to promote better coping mechanisms when dealing with stress in this population [43, 44].

In the future, it would be advantageous to extend this study to the population of non-cancer palliative healthcare professionals. Future studies could also include other instruments for assessing burnout’s risk and quality of life.

After this study, a Burnout Consultation was created at the Institution, to support professionals at risk or already affected.

### Supplementary Information

**Additional file 1.**

## Data Availability

The datasets generated during and analyzed during the current study are available from the corresponding author upon reasonable request.
