# Supplementary material for: Burnout and quality of life in Portuguese healthcare professionals working in oncology and palliative care—a preliminary study
Source: BMC Palliat Care. 2023 Oct 13;22:155. doi: 10.1186/s12904-023-01273-7 (PMC10571454; doi:10.1186/s12904-023-01273-7)
Supplement: Supplementary file 1 — Additional file 1. [file 12904_2023_1273_MOESM1_ESM.docx]

**SUPPLEMENTARY DATA**

*Maslach Burnout Inventory - MBI (Portuguese)*

| **Burnout** | **Descritivo** |
| --- | --- |
| Exaustão Emocional  (Emotional Exhaustion) | 1. Sinto-me vazio emocionalmente, por causa do meu trabalho.  2. No fim do dia de trabalho, sinto-me exausto.  3. Sinto-me fatigado quando acordo de manhã e tenho que enfrentar mais um dia de trabalho.  6. Trabalho com pessoas o dia todo é, de facto, um esforço para mim.  8. Sinto-me esgotado devido ao meu trabalho.  13. Sinto-me muito frustrado com o meu trabalho.  14. Sinto que estou a trabalhar demasiado no meu trabalho.  16. Trabalhar diretamente com pessoas coloca-me sob demasiada tensão.  20. Sinto que estou no meu limite ("fim de linha"). |
| Despersonalização  (Depersonalization) | 5. Sinto que trato alguns utentes, como se fossem objetos impessoais.  10. Tornei-me mais insensível em relação às pessoas, desde que comecei este trabalho.  11. Preocupo-me que este trabalho me esteja a "endurecer" emocionalmente.  15. De facto, não me interessa o que acontece a alguns utentes.  22. Sinto que os utentes me culpam por alguns dos seus problemas. |
| Relação pessoal  (Personal Accomplishment) | 4. Consigo compreender facilmente como os meus utentes se sentem acerca das coisas.  7. Lido muito eficazmente com os problemas dos meus utentes.  9. Sinto que estou a influenciar positivamente a vida de outras pessoas com o meu trabalho.  12. Sinto-me muito enérgico.  17. Consigo facilmente criar uma atmosfera relaxada com os meus utentes.  18. Sinto-me entusiasmado depois de trabalhar de perto com os utentes.  19. Consegui realizar muitas coisas importantes nesta profissão.  21. No meu trabalho, lido com os problemas emocionais com muita calma. |

The Maslach Burnout Inventory (CBI) consists of three dimensions (Emotional exhaustion, Depersonalization and Personal accomplishment), defined taking into account different items.

The 22 items were recorded as assuming a value between 0 and 6. The items have the following correspondence: 0 - never; 1 - a few times a year or less; 2 - once a month or less; 3 - a few times a month; 4 - once a week; 5 - a few times a week and 6 - every day.

The calculation method for each of the burnout dimensions is as follows:

* Emotional Exhaustion = sum of item 1,2,3,6,8, 13, 14, 16 and 20

* Depersonalization = sum of item 5, 10,11,15 and 22

* Personal Accomplishment = sum of item 4, 7, 9, 12, 17,18, 19 and 21

The calculation of the scores for each of the dimensions uses these values. The burnout scale can either be evaluated using continuous values that vary between 0 and 54 in EE dimension, 0 and 30 in DP dimension, 0 and 48 in the RP dimension (characterized by mean, standard deviation, medians, quartiles and extreme values). It is also possible to define three levels of burnout in each dimension:

- Emotional Exhaustion: Low (score ≤18); Medium (score 19-26); High (score ≥ 27)
- Depersonalization: Low (score ≤ 5); Medium (score 6-9); High (score ≥ 10)
- Personal Accomplishment: Low (score ≤ 33); Medium (score 34-39); High (score ≥ 40)

In this case, burnout is defined as a combination of high levels of Emotional Exhaustion and Depersonalization and low levels of Personal Accomplishment.

*WHO Abbreviated Quality of Life Assessment Instrument, WHOQOL-Bref (Portuguese)*

| **Domain** | **Facets incorporated within domains** |
| --- | --- |
| *Physical health* | 1. Pain and discomfort |
|  | 2. Energy and fatigue |
|  | 3. Sleep and rest |
|  | 9. Mobility |
|  | 10. Activities of daily living |
|  | 11. Dependence on medicinal substances and medical aids |
|  | 12. Work capacity |
| *Psychological* | 4. Positive feelings |
|  | 5. Thinking, learning, memory and concentration |
|  | 6. Self-esteem |
|  | 7. Bodily image and appearance |
|  | 8. Negative feelings |
|  | 24. Spirituality/Religion/Personal beliefs |
| *Social relationships* | 13. Personal relationships |
|  | 14. Social support |
|  | 15. Sexual activity |
| Environment | 16. Freedom, physical safety and security |
|  | 17. Home environment |
|  | 18. Financial resources |
|  | 19. Health and social care: accessibility and quality |
|  | 20. Opportunities for acquiring new information and skills |
|  | 21. Participation in and opportunities for recreation/leisure activities |
|  | 22. Physical environment (pollution/noise/traffic/climate) |
|  | 23. Transport |

Adapted from The WHOQOL Group*

* The WHOQOL Group. Development of the World Health Organization - WHOQOL-BREF quality of life assessment. Psychol Med 1998;28: 551-8.
